# Extracellular vesicle-based therapies for neurodegenerative diseases

**DOI:** 10.1515/nipt-2025-0016

**Published:** 2025-12-19

**Authors:** Guoku Hu, Christina Gogzheyan, Sudipta Panja, Susmita Sil, Howard E. Gendelman

**Affiliations:** Department of Pharmacology and Experimental Neuroscience, 12284 University of Nebraska Medical Center, Omaha, NE, 68198, USA; Department of Biological Sciences, Northern Kentucky University, Highland Heights, KY, 41099, USA

**Keywords:** extracellular vesicles, microRNA, neuroinflammation, neurodegeneration, drug delivery, colostrum

## Abstract

Extracellular vesicles (EVs) are mediators of neurodegeneration and emerging therapeutic tools for central nervous system disorders. On the one hand, they help spread beta amyloid, tau, *α*-synuclein, TDP-43, and mutant SOD1, contributing to the signs and symptoms of Alzheimer’s, Parkinson’s, Amyotrophic lateral sclerosis, and Huntington’s Diseases. By activating glial cells, they promote chronic neuroinflammation through carrying cytokines, inflammasomes, and chemokines. On the other hand, EVs’ ability to transport neuroregulatory products and cross the blood–brain barrier makes them ideal vehicles for drug delivery. Their function can be surface-modified to deliver targeted therapies, including anti-inflammatory and neuroprotective regulatory RNAs, proteins, and lipids, as well as factors that help maintain neural homeostasis. Notably, we suggest that colostrum-derived EVs, enriched with growth factors and immune-regulatory microRNAs, offer a natural, scalable, and biocompatible source for neuroprotective treatment. Although EVs can act as “Janus-faced” entities – serving both as disease initiators and versatile therapeutic vehicles – controlling their activity can enable immune-based therapeutics for neurodegenerative diseases.

## Introduction

The central nervous system (CNS) functions as a highly integrated network where precise intercellular communication is paramount for homeostasis. Dysregulation of this communication is a seminal event in the pathogenesis of neurological disease. Extracellular vesicles (EVs), including exosomes, microvesicles, and lipid-bilayer nanoparticles, have emerged as master regulators of this cellular crosstalk, facilitating the horizontal transfer of bioactive molecules – including proteins, lipids, mRNAs, and non-coding RNAs – between neurons, glia, and peripheral immune cells [1], 2]. Among their most potent cargo are microRNAs (miRNAs), small non-coding RNAs that enact post-transcriptional gene silencing by directing target mRNAs for degradation or translational repression, thereby fine-tuning complex cellular processes [3], 4]. The intrinsic biological properties of EVs – their stability in biological fluids, low immunogenicity, and unique capacity to traverse the formidable blood-brain barrier (BBB) – have propelled their exploration as next-generation drug delivery vehicles [5], 6]. These attributes make them exceptionally well-suited to addressing the entrenched challenges of delivering therapeutics to the CNS. EVs are mediators of intercellular communication. Their natural biocompatibility, limited immunogenicity, and capacity to carry diverse therapeutic payloads (proteins, lipids, and regulatory miRNAs) make them promising biomarkers and therapeutic vehicles for various neurological disorders. Their ability to cross the blood-brain barrier further enhances their potential. EVs can be used in diagnosing and treating Alzheimer’s and Parkinson’s disease, amyotrophic lateral sclerosis, Huntington’s disease, and multiple sclerosis, among others. Despite differences in their pathobiology, these diseases share common features, including chronic neuroinflammation, misfolded and aggregated proteins, and neurodegeneration. This review critically examines two innovative and complementary EV-based therapeutic strategies. The first involves engineering EVs to deliver anti-inflammatory miRNAs that can reprogram activated microglia and inhibit pro-inflammatory signals. The second employs colostrum EVs as a natural, safe, and multi-targeted treatment platform. A comprehensive review of preclinical studies highlights mechanistic links between EV-driven immune modulation and neuroprotection, including enhanced synaptic plasticity, decreased protein buildup, and improved neural recovery. We propose that these innovative methods represent a shift from merely managing symptoms to fundamentally modifying the disease process. Finally, we discuss translational barriers such as standardization, manufacturing, and regulatory challenges that must be addressed to realize the full clinical potential of EV-based therapies and nanotherapeutics.

Neurodegenerative diseases, including Alzheimer’s and Parkinson’s diseases (AD and PD), amyotrophic lateral sclerosis (ALS), Huntington’s disease (HD), and multiple sclerosis (MS), represent a devastating spectrum of conditions with diverse clinical manifestations but strikingly similar underlying pathophysiological features. These include the buildup of disease-specific misfolded proteins (Aβ peptides and tau in AD, *α*-synuclein in PD, TDP-43 in ALS, mutant huntingtin (mHTT) in HD), ongoing neuroinflammation, oxidative stress, mitochondrial dysfunction, and progressive neuronal loss [7], [8], [9], [10], [11], [12], [13], [14]. At the core of this harmful process is the persistent, maladaptive activation of microglia, the brain’s resident immune cells. Chronic microglial activation leads to a continuous release of pro-inflammatory cytokines (e.g., TNF-α, IL-1β, IL-6), reactive oxygen species (ROS), and other toxic factors that not only fail to resolve the initial damage but also promote synaptic injury, hinder protein clearance, and ultimately accelerate neuronal death [15], [16], [17]. Current pharmacological therapies for these diseases are mostly palliative, providing temporary symptomatic relief without stopping the underlying degenerative processes. A key obstacle to developing disease-modifying treatments is the BBB, which effectively blocks over 98 % of small-molecule drugs and nearly all large-molecule neurotherapeutics from entering the CNS [18], 19]. Therefore, there is a critical and unmet need for innovative therapeutic platforms that can bypass or cross the BBB to directly target core disease mechanisms, with dysregulated neuroinflammation presenting a prime and shared target. This review explores how EV-based therapeutics are uniquely positioned to meet this formidable challenge. We focus specifically on two innovative and complementary strategies: first, the use of engineered or stem cell-derived EVs loaded with anti-inflammatory miRNAs to precisely modulate microglia and other immune cells across the neurodegenerative spectrum; and second, the application of colostrum-derived EVs (C-EVs) as a natural, safe, and therapeutically rich source of multi-factor neuroprotective cargo. By critically examining the mechanistic evidence linking EV-mediated suppression of neuroinflammation to functional neuroprotection and behavioral recovery, we aim to delineate a transformative therapeutic pathway to mitigate the neuropathological processes that define these debilitating disorders.

## CNS and EVs are pathological and therapeutic vehicles

**Figure 1:**
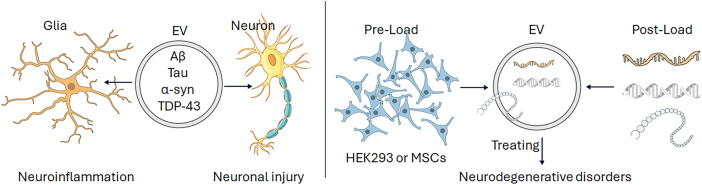
EVs and the CNS. Pathological and therapeutic vehicles. EVs serve dual roles in the CNS, acting both as mediators of neurodegenerative pathology and as potential tools for therapeutic interventions. Left panel: Pathological EVs derived from neurons, astrocytes, and microglia facilitate “prion-like” propagation of misfolded proteins, including amyloid-β (Aβ) oligomers and hyperphosphorylated tau in AD, *α*-synuclein (α-syn) aggregates in PD, and mutant SOD1 or TDP-43 in ALS. These EVs are internalized by recipient neurons or glial cells via clathrin-mediated endocytosis, macropinocytosis, or lipid raft-dependent uptake, leading to cytosolic cargo release, templated misfolding, and propagation of neurotoxicity. Additionally, EVs derived from activated microglia carry pro-inflammatory mediators (IL-1β, TNF-α, NLRP3 inflammasome components), promoting neuroinflammation and neuronal injury. Right panel: Therapeutic EVs exploit the same transport and targeting properties for beneficial purposes. Engineered EVs – derived from mesenchymal stem cells (MSCs) or HEK293 cells – can be pre-loaded through genetic modification or post-loaded with therapeutic cargos such as siRNAs, miRNAs, proteins, or small molecules. Surface functionalization with targeting ligands (RVG peptide, transferrin receptor antibodies, or CNS-specific aptamers) enhances BBB penetration and cell-type specificity. Furthermore, EVs isolated from biofluids can serve as biomarkers (EV-associated p-tau, *α*-syn, or NfL) for early diagnosis, disease monitoring, and theranostic applications in neurodegenerative disorders. Icons in this figure were adapted from the NIH BioArt collection (https://bioart.niaid.nih.gov).

The functional role of EVs is intrinsically linked to the physiological and pathological state of their parent cell. This Janus-faced duality is starkly evident in the CNS, where EVs can function as both insidious agents of disease progression and powerful therapeutic tools (Figure 1).

### EVs as pathological vectors in neurodegeneration

The “prion-like” propagation of misfolded proteins involves intercellular exchange, which is a key feature of many neurodegenerative diseases. EVs are recognized as a primary vehicle for this transmission [20], [21], [22], [23], [24], [25], [26]. Neurons and glia under proteostatic stress release EVs enriched with toxic oligomers and aggregates. In AD, EVs transport amyloid-β oligomers and hyperphosphorylated tau species, aiding the spread of pathology from the entorhinal cortex to the hippocampus and neocortex [20], 27]. In PD, EVs carry oligomeric and fibrillar *α*-synuclein, which display higher cytotoxicity and seeding ability than their intracellular forms, contributing to the characteristic progression of Lewy pathology [28], 29]. In ALS, EVs facilitate the intercellular spread of misfolded TDP-43 and mutant SOD1 between motor neurons, fueling relentless disease progression [30], 31]. In HD, EVs may contain N-terminal fragments of mHTT, potentially aiding in the dissemination of pathology beyond the striatum [32], [33], [34]. These pathological EVs are taken up by recipient cells through various endocytic pathways, such as clathrin-mediated endocytosis, macropinocytosis, or lipid raft-mediated uptake. Once inside, the cargo is released into the cytosol, seeding the aggregation of normally folded proteins and initiating a self-perpetuating cycle of misfolding and neurodegeneration [35]. The uptake process is often mediated by surface proteins on the EV, such as phosphatidylserine, which interact with receptors on target cells. Beyond promoting protein aggregation, pathological EVs worsen inflammation. EVs released by activated microglia can carry and deliver pro-inflammatory cytokines (e.g., IL-1β), bioactive lipids, and components of the NLRP3 inflammasome, triggering inflammatory activation in recipient microglia and astrocytes. This creates a feedback loop of neuroinflammation that intensifies neuronal damage and dysfunction [36], 37]. Furthermore, EVs from stressed neurons can carry complement proteins or chemokines that recruit peripheral immune cells, breaching the CNS immune privilege.

### Using EVs as therapeutic vehicles

The properties that make EVs effective as pathological vectors – such as their biological stability, ability to cross the BBB, and efficient cellular uptake – can be utilized for therapeutic purposes, representing a significant paradigm shift. As natural nanoparticles, EVs offer better biocompatibility and lower immunogenicity compared to synthetic lipid nanoparticles or viral vectors, especially when derived from autologous or immune-privileged sources like mesenchymal stem cells (MSCs). This allows for repeated administration without causing significant adverse immune reactions, which is critical for chronic neurodegenerative diseases that need long-term treatment [38], [39], [40].

While EVs have inherent targeting capabilities, their surfaces can be modified to improve targeting specificity. Genetic or chemical engineering can enable the display of targeting ligands, such as the Rabies Virus Glycoprotein (RVG) peptide for neuron targeting or antibodies against the Transferrin Receptor (TfR) for better BBB transcytosis. This facilitates precise delivery to specific CNS cell types, increasing therapeutic effectiveness while reducing off-target effects [41], 42]. Recent progress includes using CNS-specific aptamers and peptide ligands identified via phage display. A key aspect of EV therapeutics is their capacity to be loaded with various therapeutic payloads. Strategies can be broadly classified as: Pre-loading (Parent Cell Engineering): Genetic modification of parent cells (e.g., MSCs, HEK293) to overexpress desired therapeutic proteins, miRNAs, or CRISPR-Cas9 components, which are then packaged into EVs during biogenesis. This method often yields EVs with naturally sorted and functional cargo [7]. Post-loading (Direct Loading): Isolation of naive EVs followed by external loading using techniques such as electroporation, sonication, saponin permeabilization, or simple co-incubation. This allows for the incorporation of small molecules, oligonucleotides (siRNAs, miRNAs, ASOs), and recombinant proteins, offering flexibility for a wide range of therapeutics [8], [9], [10].

The same pathological EVs involved in disease propagation can be harvested from accessible biofluids like plasma, serum, and cerebrospinal fluid (CSF). The quantification and characterization of EV-associated biomarkers – such as phosphorylated tau (p-tau181, p-tau217) in AD, oligomeric *α*-synuclein in PD, or neurofilament light chain (NfL) across disorders – provide a minimally invasive “liquid biopsy” tool for early diagnosis, patient stratification, and objective monitoring of therapeutic efficacy [11], 12]. The integration of EV-based diagnostics with therapeutics (“theranostics”) represents a powerful personalized medicine approach.

## EVs target neuroinflammation

**Figure 2:**
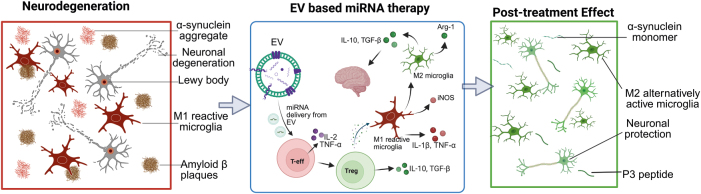
EV–based therapeutics in neurodegenerative diseases. Pathological features of neurodegeneration (left) include neuronal degeneration, protein aggregates (lewy bodies, amyloid *β* plaques, *α*-synuclein), and M1 reactive microglia. Therapeutic EVs loaded with miRNAs (center) modulate immune responses by reprogramming M1-phenotype microglia to a neuroprotective M2 phenotype and regulating T cell populations. Post-treatment effects (right) show reduced neuroinflammation and enhanced expression of neuroprotective agents (P3 peptide, *α*-synuclein monomers). The figure was generated using BioRender (https://biorender.com).

Despite diverse initial triggers – such as genetic mutations, environmental toxins, or autoimmune issues – AD, PD, ALS, HD, and MS all share a common pathway involving chronic, maladaptive neuroinflammation. This similar pathophysiology makes the brain’s immune system a crucial and logical target for broad-spectrum treatment approaches (Figure 2).

### Spectrum of microglial biology

Microglia, the CNS’s primary resident macrophages, are highly adaptable, existing across a spectrum of activation states. The classical simplistic M1/M2 dichotomy, while useful as a guiding concept, simplifies what is actually a continuous range of activation states [13]. Activated by pathogen-associated molecular patterns (PAMPs), damage-associated molecular patterns (DAMPs), or protein aggregates like amyloid beta (Aβ) and alpha synuclein (α-syn), microglia adopt an inflammatory state. This state is characterized by increased expression of surface markers (CD86, CD32, MHC-II) and vigorous secretion of pro-inflammatory mediators, including tumor necrosis factor alpha (TNF-α), interleukin-one beta (IL-1β), IL-6, reactive oxygen species (ROS), and nitric oxide (NO) [14]. Its transcriptomic signature is governed by key regulators such as NF-κB and STAT1. While this activation is essential for pathogen defense and initial damage control, prolonged activation can have harmful effects: excessive synaptic pruning via complement cascade activation, impaired neurogenesis, and direct neuron damage [15], [16], [17]. The M2 (anti-inflammatory/repair) phenotype: Induced by anti-inflammatory cytokines such as IL-4, IL-13, and IL-10, microglia shift to control anti-inflammatory state, expressing markers like CD206, Arg1, and Ym1/2. Homeostatic microglia play a central role in resolving inflammation, clearing cellular debris through enhanced phagocytosis, and fostering tissue repair by releasing neurotrophic factors (BDNF, GDNF), anti-inflammatory cytokines (IL-10, TGF-β), and pro-remyelination factors [13], 18], 19]. Recent single-cell transcriptomic research has uncovered additional diversity within the M2 state, including subsets specialized in lipid metabolism and iron sequestration. In the context of chronic neurodegeneration, a persistent imbalance – often caused by failure of resolution pathways – tends to favor a pro-inflammatory state, creating a self-perpetuating toxic environment that continuously accelerates disease progression.

### Astrocyte reactivity

Astrocytes, essential for CNS homeostasis, including synaptic maintenance, blood flow regulation, and ionic balance, undergo reactive gliosis in neurodegeneration. Similar to microglia, reactive astrocytes can adopt broadly harmful or protective states. For example, induced by the cytokine trio (IL-1α, TNF-α, C1q) released from activated M1 microglia, A1 astrocytes lose their normal homeostatic functions, including the ability to promote neuronal survival and synaptogenesis. Conversely, they gain a neurotoxic phenotype, secreting factors that directly damage neurons and oligodendrocytes and contribute to their death [43]. The presence of A1 astrocytes, characterized by a specific transcriptomic profile, is a feature of AD, PD, ALS, and MS. Induced after ischemic stroke or other insults, A2 astrocytes upregulate neurotrophic factors (BDNF, GDNF) and thrombospondins, and contribute to tissue repair and recovery [20], 43]. Therefore, a successful anti-inflammatory strategy must also aim to suppress the induction of neurotoxic A1 astrocytes by modulating the upstream microglial response, thereby restoring a healthy astrocytic population.

### Peripheral immune cells

Neuroinflammation is not confined to the CNS parenchyma alone. Strong evidence shows significant involvement of the peripheral immune system, especially in MS, and its role is increasingly recognized in AD, PD, and ALS [44]. Adaptive immune cells, including autoreactive CD4+ T lymphocytes (such as Th1 and Th17) and CD8+ cytotoxic T cells, along with B cells, can infiltrate the CNS. Additionally, systemic inflammation can prime CNS-resident microglia through cytokine signaling across the BBB [10], 45], 46]. For example, in PD, peripheral *α*-synuclein–reactive T cells have been identified [47], which further supports the bidirectional communication between the periphery and the CNS. This complex interaction highlights the need for therapeutic strategies that target both the central and peripheral immune responses to achieve comprehensive efficacy.

## Anti-inflammatory EVs and their cargo for neurodegenerative diseases

The therapeutic effectiveness of EVs is greatly influenced by their cellular origin and molecular content. A particularly promising strategy involves engineering EVs to function as targeted delivery vehicles for anti-inflammatory miRNAs, enabling precise intervention in the neuroinflammatory core of neurodegeneration with minimal off-target effects.

### miRNA EV cargo for neurodegenerative disorders

The therapeutic effectiveness of EVs heavily depends on their cellular origin and molecular content. A particularly promising strategy involves engineering EVs to serve as targeted delivery vehicles for anti-inflammatory miRNAs, enabling precise intervention in neuroinflammation associated with neurodegeneration with minimal off-target effects. The dynamic polarization of microglia and other immune cells is governed by complex transcriptional and post-transcriptional networks, which can be strategically modulated by EV-delivered miRNAs. These miRNAs can target multiple points within a pathway simultaneously, providing a powerful multi-targeted approach. For example, miR-146a is a key regulator of innate immunity [48]. miRNA acts as a critical negative feedback regulator of Toll-like receptor (TLR) and IL-1 receptor signaling pathways. It directly targets and downregulates key adapter proteins IRAK1 and TRAF6, leading to suppression of the downstream NF-κB signaling cascade. EVs loaded with miR-146a can thus effectively inhibit the production of NF-κB-driven pro-inflammatory cytokines like TNF-α, IL-1β, and IL-6, which are chronically elevated in AD, PD, and ALS [48], [49], [50]. This intervention breaks the cycle of inflammation-driven neurodegeneration at a central signaling hub. miR-124 is critical for conferring CNS-specific phenotypes. As the most abundant miRNA in the mature CNS, miR-124 is essential for maintaining microglial quiescence and promoting a neuroprotective phenotype. It represses transcription factors such as C/EBPα and PU.1, which are master regulators of M1 gene expression. Studies demonstrate that EVs enriched with miR-124 effectively shift microglia from a pro-inflammatory to an anti-inflammatory state [42], 51]. This shift is functionally coupled with enhanced phagocytic clearance of Aβ and *α*-synuclein aggregates and stimulated release of neurotrophic factors like BDNF and GDNF [52], [53], [54]. miR-21 is an anti-apoptotic and anti-inflammatory mediator. It exerts its neuroprotective effects through a multi-faceted mechanism. By targeting PTEN (Phosphatase and Tensin Homolog), it alleviates inhibition of the PI3K/Akt signaling pathway, a critical pro-survival cascade in neurons. Additionally, its anti-apoptotic role through PDCD4 suppression and its influence on T-cell responses and microglial activation contribute to an overall anti-inflammatory and pro-survival microenvironment, as demonstrated in models of MS and spinal cord injury [55], 56].

### Therapeutic applications for EVs in neurodegenerative diseases

The versatility and efficacy of miRNA-loaded EVs are supported by a growing body of compelling preclinical evidence across numerous neurodegenerative models. Table 1 offers a detailed summary of key studies [57], [58], [59], [60], [61], [62], [63]. In addition to the studies in Table 1, other approaches have shown promise. For example, EVs engineered to carry neprilysin, an Aβ-degrading enzyme, have been shown to reduce plaque burden [64], 65]. Combining anti-inflammatory miRNA delivery with such clearance strategies presents a powerful multi-pronged approach to AD pathology. Besides miR-124, EVs have been utilized to deliver GDNF, a potent neurotrophic factor for dopaminergic neurons. Macrophage-derived EVs engineered to release GDNF demonstrated significant neuroprotection and functional recovery in PD models, highlighting the EV platform’s flexibility to deliver protein cargo alongside miRNAs [66]. The application in ALS is particularly crucial given the lack of effective treatments. The ability of miR-124-EVs to modulate the inflammatory environment of the spinal cord and directly protect motor neurons offers a novel disease-modifying strategy [67]. Further research is exploring EVs loaded with miRNAs targeting TDP-43 pathology itself. While direct EV-miRNA studies are limited, the strong inflammatory component in HD, driven by mHTT in microglia and astrocytes, provides a clear rationale. EVs loaded with miR-146a could suppress the elevated IL-6 and other inflammatory mediators in the striatum [68]. Furthermore, EVs could be explored to deliver silencing RNAs targeting the mHTT mRNA itself. The dual approach demonstrated in MS models – simultaneously promoting remyelination (miR-219/338) and suppressing neuroinflammation (miR-124) – exemplifies the capacity of EV-based therapy to address the complex, multi-factorial pathologies of neuro-immune disorders [42], 69]. This surpasses the capability of single-target drug therapies.

**Table 1:** miRNA-Loaded EVs in neurodegenerative models.

Disease model	EV source/Type	Key miRNA cargo	Administration route	Key findings & mechanisms	Reference
Alzheimer’s disease (AD)	MSC-EVs	miR-146a	Intravenous	Attenuated neuroinflammation, reduced synaptic loss, and improved cognitive function in morris water maze.	[57]
AD	SH-SY5Y-derived EVs	miR-124–3p	AD triculture (microglia–neurons–astrocytes)	Counteracted neuronal apoptosis, halted astrocyte morphological and immune dysregulation.	[58]
Parkinson’s disease (PD)	hUCB-MNC-EVs	miR-124–3p	Intracerebroventricular	Promoted neuroprotection and counteracted PD-related motor deficits in the 6-OHDA mouse model.	[59]
PD	ADSC-EVs	miR-188–3p	Intravenous	Suppressed the levels of CDK5 and NLRP3 and alleviated substantia nigra damage in MPTP-induced PD mice.	[60]
Amyotrophic lateral sclerosis	MSC-EVs	miR-29 b-3p	Human iAstrocytes from patients, and SOD1G93A mice astrocytes	Rescued motor neuron survival via antioxidant effects (NQO1 upregulation) and reduced ROS.	[61]
Multiple sclerosis (MS)	HEK293T cell-EVs	miR-219	Intranasal	Promoted myelin regeneration and decreased clinical scores in the EAE model.	[62]
Huntington’s disease (HD)	HEK293 cell-EVs	miR-124–3p	Bilateral stereotaxic injections into the striatum	Reduced the expression of REST (an HD-relevant target).	[63]

MSC, mesenchymal stem cell; hUCB-MNC, human umbilical cord blood mononuclear cell; ADSC, adipose-derived stem cell; 6-OHDA, 6-hydroxydopamine; EAE, experimental autoimmune encephalomyelitis.

This collective body of evidence firmly establishes miRNA-cargo EVs not as a generic therapeutic, but as a highly versatile and tunable platform that can be tailored to address the distinct immunopathological signature of each neurodegenerative disorder. The choice of EV source, miRNA cargo, and administration route can be optimized for specific disease contexts.

## Colostrum EVs (C-EVs) are a natural, multi-targeted therapeutic

While engineered EVs offer the appeal of precision, C-EVs represent a “nature-engineered” therapeutic platform that combines an excellent safety profile with inherent, multi-targeted bioactivity, offering a complementary and highly translatable approach, especially for broad-spectrum neuroprotection.

### Composition and innate col-EVs bioactivity

Bovine colostrum, the first milk produced postpartum, is a biofluid evolutionarily optimized for immune protection and tissue development in the neonate. EVs isolated from colostrum effectively encapsulate and protect this innate bioactivity, creating a natural therapeutic cocktail.

C-EVs are enriched with a diverse array of immunomodulatory miRNAs, such as miR-30 b, miR-148a, and miR-155, known to play critical roles in fine-tuning immune cell development, differentiation, and function, especially during the transition from an intrauterine to a pro-inflammatory external environment [70]. This provides a natural, broad-spectrum regulatory RNA network capable of simultaneously influencing multiple inflammatory pathways, potentially acting together to produce a balanced immune response. The proteomic profile of C-EVs is filled with well-known bioactive factors. For example, lactoferrin is a multifunctional glycoprotein with strong anti-inflammatory, antimicrobial, and iron-chelating properties. It has been shown to inhibit microglial activation by blocking NF-κB translocation, reduce Aβ-induced neurotoxicity, and exert neuroprotective effects in AD models. Its iron-chelating property may also mitigate oxidative stress [71], [72], [73]. Transforming Growth Factor-Beta (TGF-β) is a master anti-inflammatory cytokine that promotes the expansion and function of regulatory T cells (Tregs) and drives microglia and macrophages toward an M2-polarized, repair-oriented state [34]. Insulin-like Growth Factor-1 (IGF-1) and Fibroblast Growth Factors (FGFs) are potent neurotrophic factors that support neuronal survival, axonal growth, synaptogenesis, and oligodendrocyte viability and maturation, crucial for both neuronal health and repair of damaged myelin in diseases like MS [74], 75].

The EV membrane consists of lipids like sphingomyelin, which can promote neurite outgrowth, and phosphatidylserine, which can mediate immunomodulatory “eat-me” signals [76], [77], [78]. These components can also participate in cell signaling and influence the stability, targeting, and uptake of the vesicles.

### C-EVs preclinical models

The therapeutic potential of C-EVs is supported by a growing body of preclinical data that involves multiple mechanisms of action. For example, orally administered C-EVs have shown efficacy in reducing inflammation in colitis models, suggesting the potential to lessen systemic inflammation that can, in turn, worsen neuroinflammation [79]. This oral bioavailability is a notable practical benefit. A key study identified a buffalo colostrum-derived peptide that improved PD pathophysiology by inhibiting Cullin-3 (CUL3) [80]. CUL3 is a core part of the Keap1-CUL3 E3 ubiquitin ligase complex, which continuously targets the transcription factor Nrf2 for degradation via the proteasome. Inhibiting CUL3 stabilizes Nrf2, allowing it to move to the nucleus where it activates the Antioxidant Response Element (ARE), leading to the coordinated upregulation of a battery of over 250 cytoprotective genes involved in antioxidant defense, detoxification, and anti-inflammation [81]. It is highly plausible that this or similar bioactive peptides are naturally packaged within C-EVs, positioning them as vehicles for delivering this potent, coordinated antioxidant and anti-inflammatory strategy, which is relevant across nearly all neurodegenerative diseases. These studies suggest that C-EVs are remarkably stable and can survive passage through the gastrointestinal tract, entering the systemic circulation to exert effects on remote organs, including the CNS. This is highly relevant given the prominent role of the gut-brain axis in PD and AD.

### Manufacturing and regulatory advantages

From a translational perspective, C-EVs offer distinct practical benefits that can significantly accelerate their path to the clinic. Colostrum is a renewable, abundant, and cost-effective raw material. Established industrial-scale dairy processing, filtration, and purification technologies can be readily adapted to consistently, reproducibly, and at scale isolate C-EVs, overcoming a major production and standardization hurdle that challenges the field of cell culture-derived EV therapeutics [82]. This enables the production of clinical-grade material in quantities necessary for widespread therapeutic application. Additionally, bovine colostrum has a long history of human consumption and is designated as GRAS by the U.S. FDA [83]. Its well-established safety profile greatly simplifies the regulatory pathway for clinical development compared to novel synthetic nanoparticles or complex cell-based products, potentially reducing the time, cost, and regulatory uncertainty involved in translational research.

## Linking EV-mediated immunomodulation to neuroprotection

The ultimate goal of a disease-modifying therapy is not merely to suppress pathological processes but to actively foster an environment conducive to neuronal survival, repair, and functional restoration. EV-based therapies, particularly those leveraging specific miRNA cargo or the multifaceted cargo of C-EVs, create this critical causal link between immunomodulation and neuroprotection through a sequential, reinforcing cascade [41], 84]. Anti-inflammatory miRNAs (e.g., miR-146a, miR-124) delivered via EVs directly reprogram microglia, shifting the population balance from a cytotoxic to a protective phenotype [85], [86], [87]. This is achieved by silencing key pro-inflammatory transcripts. Concurrently, C-EVs, through their inherent protein cargo (TGF-β, Lactoferrin) [88], 89], exert a broad anti-inflammatory influence on both microglia and astrocytes, suppressing the drivers of the A1 neurotoxic astrocyte phenotype. This phenotypic switch dampens the chronic inflammatory milieu, reducing the sustained release of cytokines (TNF-α, IL-1β) and radicals (ROS, NO) that directly damage synapses, induce neuronal apoptosis, and inhibit neurogenesis. The “cooling” of the inflammatory environment is a prerequisite for repair.

The resolved inflammatory environment, now permissive for repair, is actively augmented by the EV cargo. This includes: a) the delivery of neurotrophic factors (BDNF, GDNF, IGF-1 from MSC-EVs and C-EVs) that directly support neuronal health and synaptic function; b) the activation of endogenous antioxidant defenses through the Nrf2 pathway (by C-EVs), reducing oxidative damage; and c) facilitating autophagy and proteostatic mechanisms results in the clearance of toxic protein aggregates. The synergistic outcome of suppressed inflammation and active neurosupport is the preservation of synaptic integrity, promotion of neurite outgrowth, and potentially, stimulation of neurogenesis in select brain regions. At the behavioral level, this translates into measurable functional improvements in disease models including enhanced cognitive performance in AD (e.g., Morris water maze, novel object recognition), restored motor coordination in PD and ALS (e.g., rotarod, beam walking), and improved functional scores in MS [90], [91], [92], [93], [94], [95], [96]. The correlation between molecular and functional (toxic and trophic) outcomes solidifies cause-and-effect relationships.

This integrated framework positions EVs not as mere passive delivery vessels, but as sophisticated biological systems that actively engage and recalibrate the body’s own repair and maintenance mechanisms. By simultaneously silencing pro-inflammatory signals and amplifying pro-survival pathways, EV-based strategies address the multifaceted, interactive nature of neurodegeneration in a way that single-target pharmacological agents cannot, offering a holistic therapeutic approach.

## Challenges, standardization, and future directions

Despite the compelling preclinical evidence and considerable enthusiasm, the translational pathway for EV-based therapies from the laboratory to the clinic is fraught with technical, manufacturing, and regulatory challenges that need be addressed by academia and industry regulators.

### EV production and characterization

A primary obstacle is the lack of universal standardization, leading to significant heterogeneity in EV preparations that confounds inter-study comparisons and impedes clinical development and regulatory approval. Common techniques like differential ultracentrifugation, size-exclusion chromatography, and polymer-based precipitation co-isolate heterogeneous EV subpopulations (e.g., small exosomes, larger microvesicles) and non-vesicular contaminants (e.g., lipoproteins, protein aggregates), resulting in preparations with varying purity, size distributions, and cargo profiles [97], [98], [99], [100], [101], [102]. The field is urgently moving towards implementing Good Manufacturing Practice (GMP)-compatible, scalable, and reproducible isolation methods, such as tangential flow filtration (TFF) combined with chromatography, for clinical-grade production [103], [104], [105].

Adherence to the Minimal Information for Studies of EV guidelines is paramount for rigorous characterization [43], [106], [107], [108]. This must include multi-parametric analysis: nanoparticle tracking analysis (NTA) or tunable resistive pulse sensing (TRPS) for particle concentration and size distribution; Western blot or flow cytometry (often on a nano-flow cytometer) for detection of EV-enriched transmembrane (CD63, CD81, CD9) and cytosolic (TSG101, Alix) proteins, and absence of negative markers (e.g., calnexin for endoplasmic reticulum, apolipoproteins); and transmission electron microscopy (TEM) for morphological assessment. Advanced omics (proteomics, RNA-seq) are increasingly used for in-depth cargo profiling.

### EV cargo loading and engineering

While engineering strategies exist, their efficiency, scalability, and impact on EV integrity require further optimization. Techniques like electroporation, while widely used, can induce EV aggregation, cargo precipitation, or membrane damage, reducing yield and functionality [44], 45], 109], 110]. Developing gentler, more efficient loading methods (e.g., using freeze-thaw cycles, saponin permeabilization, or novel transfection agents) that maximize cargo encapsulation without compromising EV integrity and biological activity is a critical research focus [46], 47], 111], 112].

The genetic engineering of parent cells (e.g., MSCs, HEK293) to produce designer EVs is a common pre-loading strategy. However, scaling up these engineered cell lines under defined, serum-free, and GMP-compliant conditions presents substantial challenges in ensuring batch-to-batch consistency, potency, and safety. Bioreactor technologies and advanced cell culture systems are being developed to address this.

### Pharmacokinetics, biodistribution, and dosing

Fundamental questions regarding the *in vivo* journey of therapeutic EVs remain partially unanswered and are essential for rational clinical trial design.

After systemic or intranasal administration, what proportion of the administered EV dose actually reaches the target brain region and specific cell type (e.g., neuron vs. microglia)? Advanced *in vivo* imaging techniques using radiolabeled (e.g., with Zirconium-89) or fluorescently labeled EVs, coupled with ex vivo tissue analysis using highly sensitive assays, are crucial for quantifying biodistribution and validating the efficacy of targeting strategies.

The plasma half-life, tissue accumulation, metabolism, and ultimate clearance pathways (e.g., hepatic, splenic) of exogenous EVs are not fully elucidated. Understanding these parameters is vital for determining optimal dosing intervals and predicting potential organ-specific toxicities.

Establishing the therapeutic window, determining the optimal dose (based on particle number, protein content, or cargo quantity), frequency of administration, and identifying the most effective treatment window (early prophylactic vs. late interventional) require extensive, well-designed preclinical studies in multiple, clinically relevant animal models that recapitulate key aspects of human disease.

### Regulatory and safety considerations

Navigating the regulatory landscape is a pivotal step for clinical translation. While EVs are generally well-tolerated, thorough preclinical toxicology studies are mandatory to rule out potential off-target effects, pro-thrombotic activity, unintended immunostimulation (especially with allogeneic sources), or long-term sequelae. This includes studies in immunocompetent animals and assessment of potential tumorigenicity if using stem cell-derived EVs.

Regulatory agencies (FDA, EMA) are in the process of developing specific guidelines for EV-based products, which are currently evaluated under a hybrid of existing frameworks for biologics, cell-based therapies, and advanced medicinal products. The establishment of clear, predictable, and product-class-specific regulatory pathways is essential to de-risk the development process and attract sustained industrial investment [113]. Defining critical quality attributes (CQAs) for EV products is a key part of this process.

### Concluding perspectives

While this review primarily focuses on EV-based therapies for neurodegenerative diseases, it is important to address the increasingly recognized systemic effects and peripheral roles of therapeutic EVs. EVs naturally traffic throughout the body, facilitating extensive inter-organ communication and impacting various peripheral systems. For instance, administered EVs can influence the peripheral immune response by interacting with immune cells such as lymphocytes and macrophages, potentially reducing systemic inflammation – a factor often linked to the initiation or progression of neurodegeneration [1]. Additionally, therapeutic EVs derived from Mesenchymal Stem Cells (MSCs) are already being studied for their therapeutic effects on other organ systems, including cardiovascular and metabolic systems, demonstrating their broad biodistribution and capacity for systemic therapeutic action [114], 115]. Recognizing this systemic activity is crucial, as it necessitates a careful assessment of potential off-target effects, while simultaneously allowing researchers to harness modulation of peripheral pathology, which can lead to synergistic CNS therapeutic benefits. The future of EV therapeutics is not merely promising but is on the verge of a transformative breakthrough, moving from basic research to clinical innovation. Building on current successes in understanding EV biology, the field is now actively addressing several key frontiers that could revolutionize the treatment of neurodegenerative diseases – shifting the focus from just alleviating symptoms to actually modifying the disease. These important areas of progress, which highlight the vast potential of EVs for CNS disease, are summarized in Table 2.

**Table 2:** Key frontiers shaping EV therapeutics.

Frontier	Strategy/Focus area	Primary impact/Goal
Personalized therapy	Leveraging patient-specific iPSC-derived EVs or tailoring miRNA cargo based on genetic/inflammatory profiles from liquid biopsies.	Maximizing efficacy and minimizing side effects through precision medicine.
Combination strategies	Using EVs as an adjunct to enhance standard-of-care treatments (e.g., levodopa, anti-abeta immunotherapies).	Enhancing symptomatic relief while providing neuroprotection and disease modification; delivering therapies across the BBB.
“Smart” & hybrid EVs	Developing EVs that release cargo in response to disease microenvironment cues (e.g., MMP levels, ROS, pH changes).	Ensuring spatiotemporal precision; reducing off-target effects; combining advantages of natural and synthetic delivery.
EV biology & design	Integrated proteomic, lipidomic, and transcriptomic analyses of EVs.	Identifying novel cargo and mechanisms; developing more potent “designer” EV therapeutics.

## Authorship

Guoku Hu, Christina Gogzheyan, Sudipta Panja and Susmita Sil contributed to writing, reviewing, and editing the original draft. Howard E. Gendelman contributed to conceptualization, writing, reviewing, editing, and supervision. All authors have read and approved the final manuscript.
